# Supplementary material for: SV40 Large T antigen inhibits the host serine protease FAM111A through a zinc-dependent, cleavage-avoiding mechanism
Source: bioRxiv. 2026 Aug 10:2026.08.07.743322. Preprint. [Version 1] doi: 10.64898/2026.08.07.743322 (PMC13640560; doi:10.64898/2026.08.07.743322)
Supplement: Supplementary file 1 [file NIHPP2026.08.07.743322v1-supplement-1.pdf]

bioRxiv preprint doi: https://doi.org/10.64898/2026.08.07.743322; this version posted August 10, 2026. The copyright holder for this preprint (which was not certified by peer review) is the author/funder, who has granted bioRxiv a license to display the preprint in perpetuity. It is made available under a CC-BY 4.0 International license.

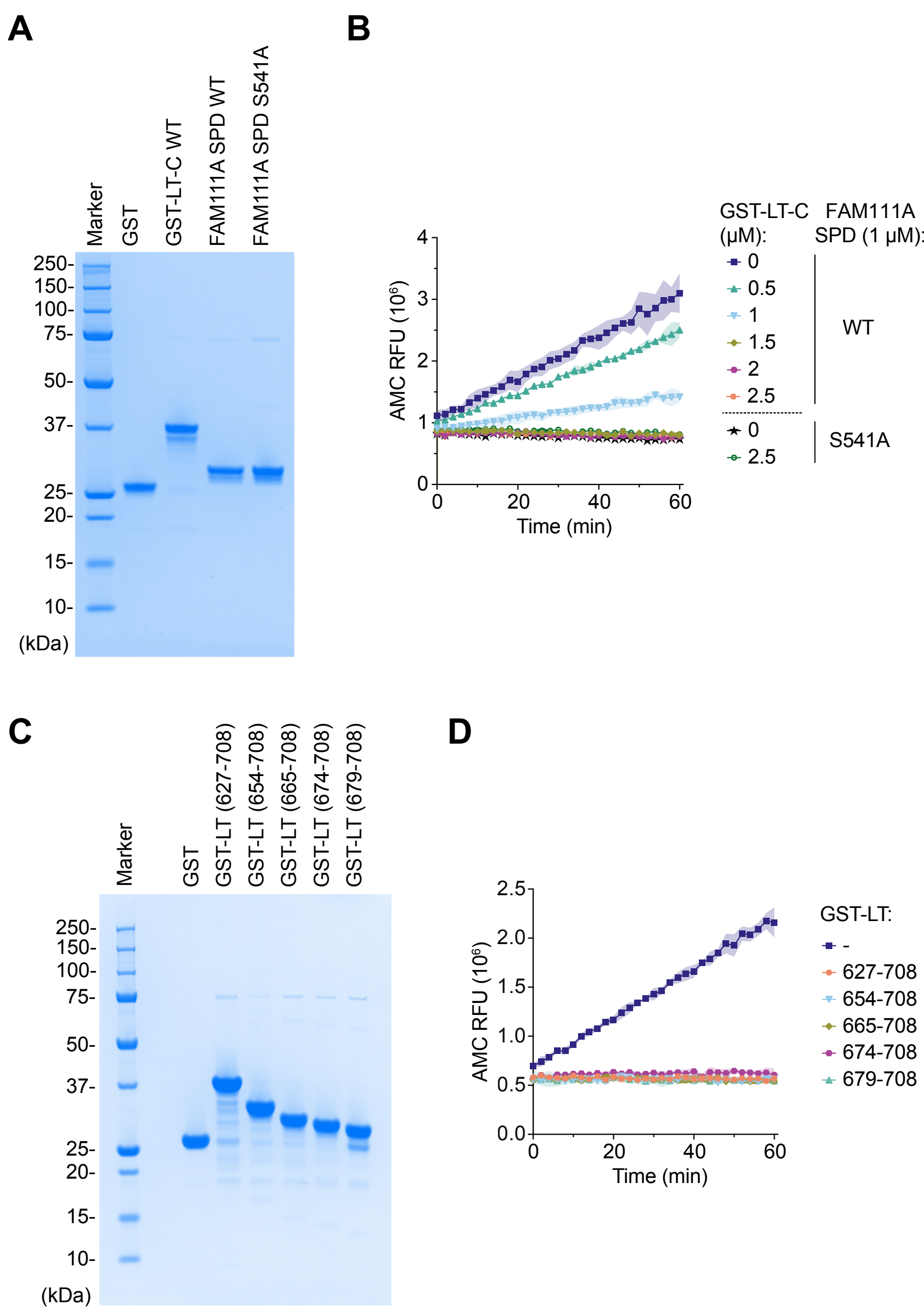

Supplementary Figure 1

bioRxiv preprint doi: https://doi.org/10.64898/2026.08.07.743322; this version posted August 10, 2026. The copyright holder for this preprint (which was not certified by peer review) is the author/funder, who has granted bioRxiv a license to display the preprint in perpetuity. It is made available under a CC-BY 4.0 International license.

Supplementary Figure 1. Purification and activity of recombinant FAM111A SPD and GST-LT-C proteins.

(A) One microgram of purified recombinant GST, GST-LT-C (WT), FAM111A serine protease domain (SPD; WT) and FAM111A SPD (S541A) analyzed by SDS-PAGE and visualized by Coomassie staining. Molecular weight markers are shown on the left.

(B) Time course of *in vitro* FAM111A SPD activity in the presence of increasing concentrations of GST-LT-C. Reactions without GST-LT-C contain GST as a control. Peptidase activity was measured using Suc-AAPF-AMC as a substrate and fluorescence from released AMC was measured over time for 1 hr at 37°C. Values represent the mean of three technical replicates, and standard deviation is indicated by the shaded area. RFU: relative fluorescence units.

(C) Two micrograms of purified recombinant GST and the indicated GST-LT proteins analyzed by SDS-PAGE. Molecular weight markers are shown on the left.

(D) Time course of *in vitro* FAM111A SPD activity in the presence of GST-LT truncation proteins. Reactions without GST-LT contain GST as a control. Peptidase activity was measured as in (B).

bioRxiv preprint doi: https://doi.org/10.64898/2026.08.07.743322; this version posted August 10, 2026. The copyright holder for this preprint (which was not certified by peer review) is the author/funder, who has granted bioRxiv a license to display the preprint in perpetuity. It is made available under a CC-BY 4.0 International license.

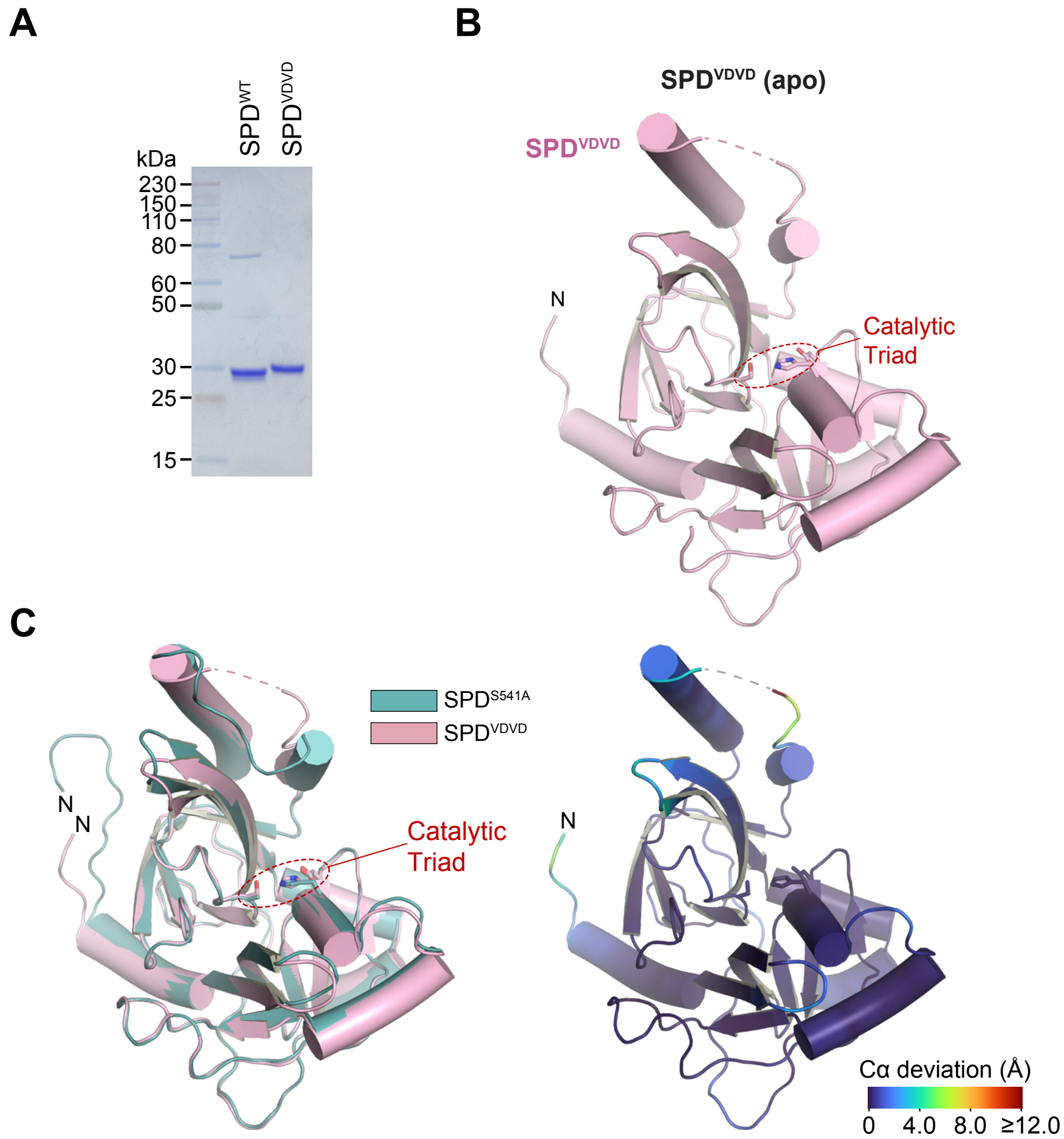

Supplementary Figure 2

bioRxiv preprint doi: https://doi.org/10.64898/2026.08.07.743322; this version posted August 10, 2026. The copyright holder for this preprint (which was not certified by peer review) is the author/funder, who has granted bioRxiv a license to display the preprint in perpetuity. It is made available under a CC-BY 4.0 International license.

Supplementary Figure 2. Purification and structural characterization of FAM111A SPD^{VDVD} (apo).

(A) 2.4 micrograms of purified recombinant FAM111A serine protease domain (SPD^{VDVD}; V347D/V351D) analyzed by SDS-PAGE and visualized by Coomassie staining. Molecular weight markers are shown on the left.

(B) Overall structure of the apo SPD^{VDVD} crystal structure shown as a cartoon representation. The catalytic triad is highlighted.

(C) Superposition of the apo SPD^{VDVD} (pink) and previously reported SPD^{S541A} (teal) structures. Left, structural overlay. Right, Cα deviation mapped onto the SPD^{VDVD} structure, with colors indicating the magnitude of local structural differences.

bioRxiv preprint doi: https://doi.org/10.64898/2026.08.07.743322; this version posted August 10, 2026. The copyright holder for this preprint (which was not certified by peer review) is the author/funder, who has granted bioRxiv a license to display the preprint in perpetuity. It is made available under a CC-BY 4.0 International license.

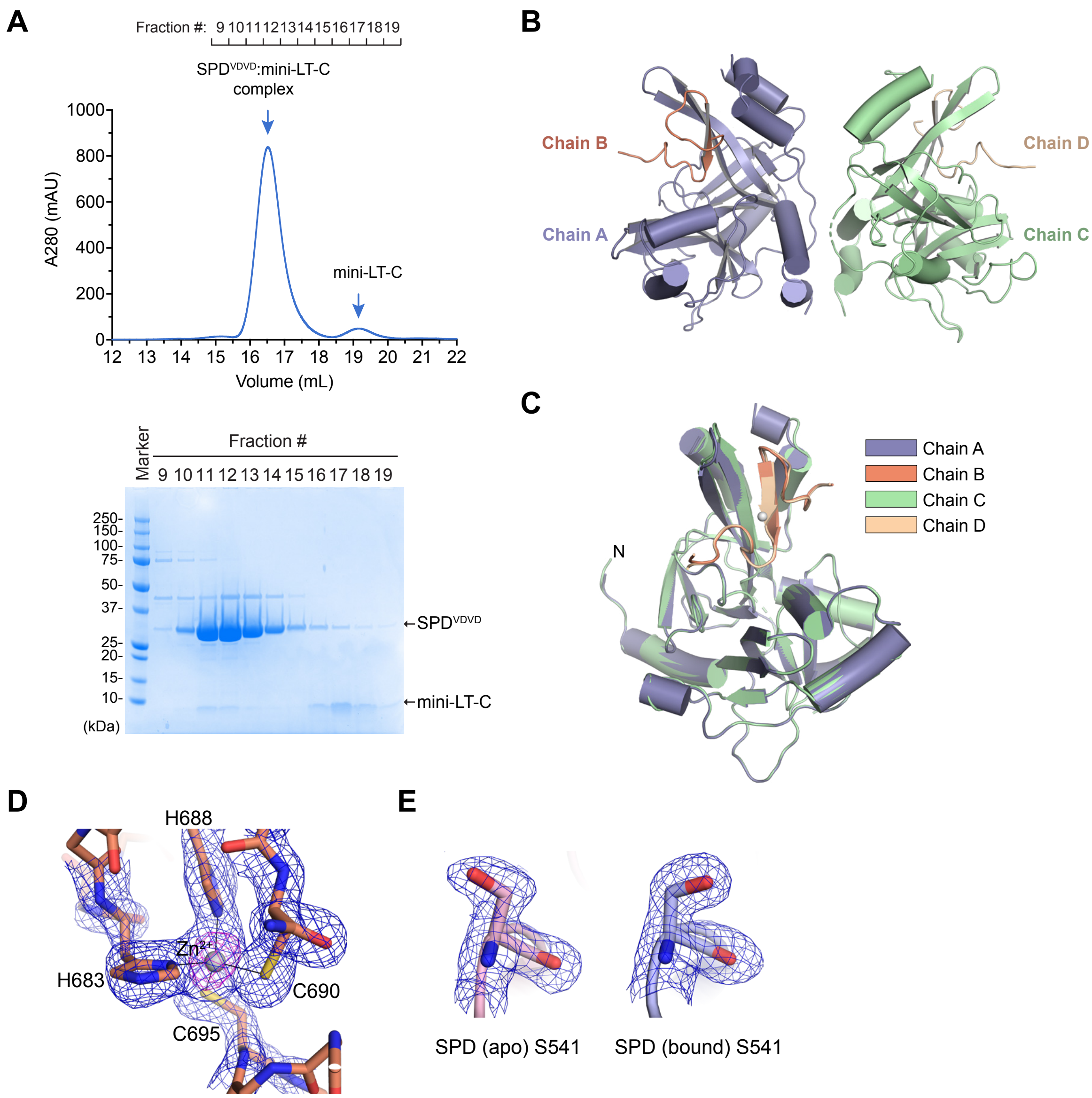

Supplementary Figure 3

bioRxiv preprint doi: https://doi.org/10.64898/2026.08.07.743322; this version posted August 10, 2026. The copyright holder for this preprint (which was not certified by peer review) is the author/funder, who has granted bioRxiv a license to display the preprint in perpetuity. It is made available under a CC-BY 4.0 International license.

Supplementary Figure 3. Purification and structural characterization of the SPD^{VDVD}:mini-LT-C complex.

(A) Purification of the SPD^{VDVD}:mini-LT-C complex by size-exclusion chromatography. Top, elution profile showing the major protein peak used for crystallization. Bottom, Coomassie-stained SDS-PAGE analysis of the indicated fractions. The positions of SPD^{VDVD} and mini-LT-C are indicated on the right, and molecular weight markers are shown on the left.

(B) Overall structure of the asymmetric unit of the SPD^{VDVD}:mini-LT-C crystal. The asymmetric unit contains two SPD^{VDVD}:mini-LT-C complexes (chains A/B and C/D).

(C) Superposition of the two SPD^{VDVD}:mini-LT-C complexes in the asymmetric unit. Chains are colored as indicated.

(D) 2mFo-DFc electron density map (blue mesh, contoured at 1σ) and anomalous difference map (magenta mesh, contoured at 5σ) depicting the zinc-binding site of mini-LT-C. The zinc ion and coordinating residues His683, His688, Cys690, and Cys695 are shown as sticks.

(E) 2mFo-DFc electron density map contoured at 1σ showing density for the catalytic Ser541 in the apo SPD^{VDVD} structure (left) and the SPD^{VDVD}:mini-LT-C complex (right).

bioRxiv preprint doi: https://doi.org/10.64898/2026.08.07.743322; this version posted August 10, 2026. The copyright holder for this preprint (which was not certified by peer review) is the author/funder, who has granted bioRxiv a license to display the preprint in perpetuity. It is made available under a CC-BY 4.0 International license.

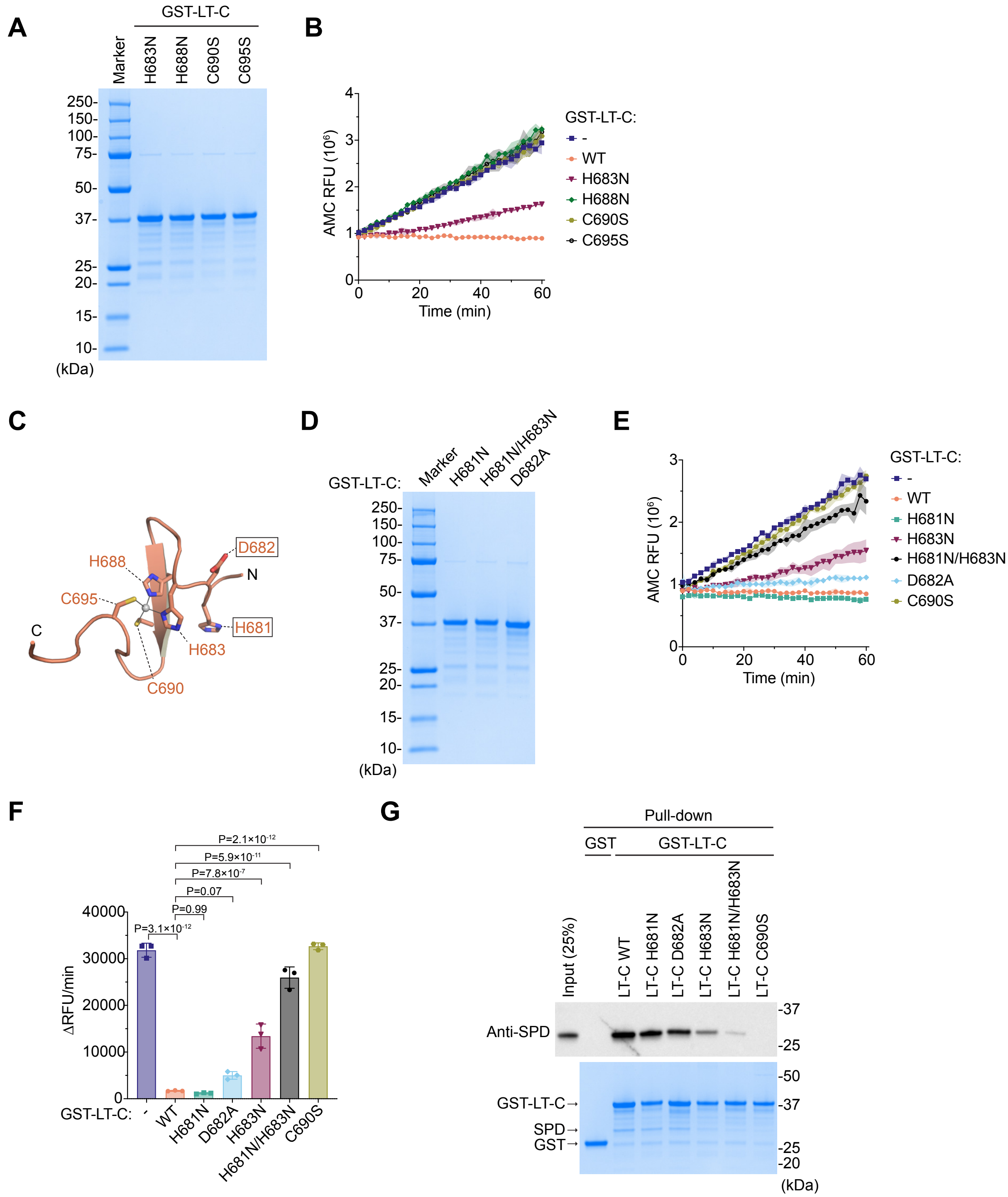

Supplementary Figure 4

bioRxiv preprint doi: https://doi.org/10.64898/2026.08.07.743322; this version posted August 10, 2026. The copyright holder for this preprint (which was not certified by peer review) is the author/funder, who has granted bioRxiv a license to display the preprint in perpetuity. It is made available under a CC-BY 4.0 International license.

Supplementary Figure 4. Effects of zinc-binding motif mutations on LT-C-mediated inhibition of and binding to FAM111A.

(A) One microgram of purified recombinant GST-LT-C zinc-binding motif mutants (H683N, H688N, C690S, and C695S) analyzed by SDS-PAGE and visualized by Coomassie staining. Molecular weight markers are shown on the left.

(B) Time course of *in vitro* FAM111A SPD activity in the presence of GST-LT-C (wild-type or zinc-binding motif mutants H683N, H688N, C690S, and C695S). Reactions without GST-LT-C contain GST as a control. Peptidase activity was measured using Suc-AAPF-AMC as a substrate and fluorescence from released AMC was measured over time for 1 hr at 37°C. Values represent the mean of three technical replicates, and standard deviation is indicated by the shaded area. RFU: relative fluorescence units.

(C) Close-up view of the LT-C zinc-binding motif. Side chains of residues H681 and D682, in addition to the zinc-coordinating residues (H683, H688, C690, and C695) are shown as sticks.

(D) One microgram of purified recombinant GST-LT-C mutants (H681N, H681N/H683N, and D682A) analyzed by SDS-PAGE and visualized by Coomassie staining.

(E) Time course of in vitro FAM111A SPD activity in the presence of GST-LT-C, either wild-type or mutants (H681N, H681N/H683N, and D682A). Reactions without GST-LT-C contain GST as a control. Peptidase activity was measured as in (B). Values represent the mean of three technical replicates, and standard deviation is indicated by the shaded area.

(F) In vitro protease activity of FAM111A SPD measured in the presence of GST-LT-C (wild-type or mutants H681N, H683N, H681N/H683N, D682A, and C690S). Reactions without GST-LT-C contain GST as a control. Values are mean ± SD of three technical replicates. Statistical significance was determined by one-way ANOVA with Dunnett's multiple-comparison test.

(G) In vitro GST pull-down assay of FAM111A SPD with GST-LT-C (wild-type, or mutants H681N, H683N, H681N/H683N, D682A, and C690S). Bound FAM111A SPD was detected by western blotting (top), and GST proteins were visualized by Coomassie staining (bottom). Molecular weight markers are shown on the right.

bioRxiv preprint doi: https://doi.org/10.64898/2026.08.07.743322; this version posted August 10, 2026. The copyright holder for this preprint (which was not certified by peer review) is the author/funder, who has granted bioRxiv a license to display the preprint in perpetuity. It is made available under a CC-BY 4.0 International license.

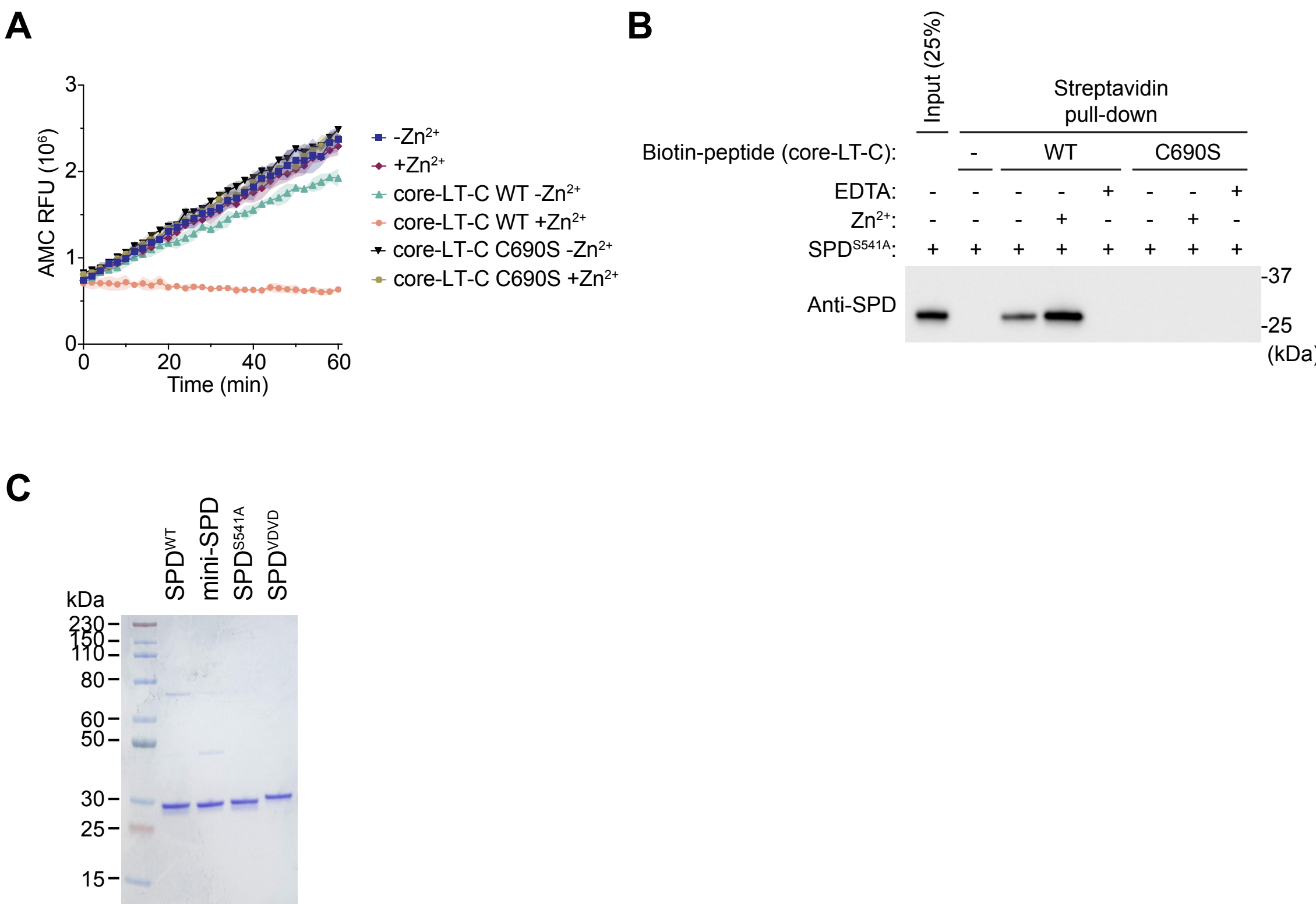

Supplementary Figure 5

bioRxiv preprint doi: https://doi.org/10.64898/2026.08.07.743322; this version posted August 10, 2026. The copyright holder for this preprint (which was not certified by peer review) is the author/funder, who has granted bioRxiv a license to display the preprint in perpetuity. It is made available under a CC-BY 4.0 International license.

Supplementary Figure 5. Zinc is required for LT-C-mediated inhibition and interaction with FAM111A.

(A) Time course of *in vitro* FAM111A SPD activity measured in the presence of a synthetic LT-C peptide corresponding to residues 679-704 (wild-type or C690S) incubated with or without $ZnCl_2$. Peptidase activity was measured using Suc-AAPF-AMC as a substrate and fluorescence from released AMC was measured over time for 1 hr at 37°C. Values represent the mean of three technical replicates, and standard deviation is indicated by the shaded area. RFU: relative fluorescence units.

(B) *In vitro* streptavidin pull-down assay using biotinylated LT-C peptides corresponding to residues 679-704 (wild-type or C690S) incubated with FAM111A SPD S541A in the presence or absence of a twofold molar excess of $ZnCl_2$. Where indicated, EDTA was added to the reaction. Bound FAM111A SPD was detected by western blotting. Molecular weight markers are shown on the right.

(C) The indicated purified recombinant SPD proteins (1.2 µg each) were analyzed by SDS-PAGE and visualized by Coomassie staining. Molecular weight markers are shown on the left.

bioRxiv preprint doi: https://doi.org/10.64898/2026.08.07.743322; this version posted August 10, 2026. The copyright holder for this preprint (which was not certified by peer review) is the author/funder, who has granted bioRxiv a license to display the preprint in perpetuity. It is made available under a CC-BY 4.0 International license.

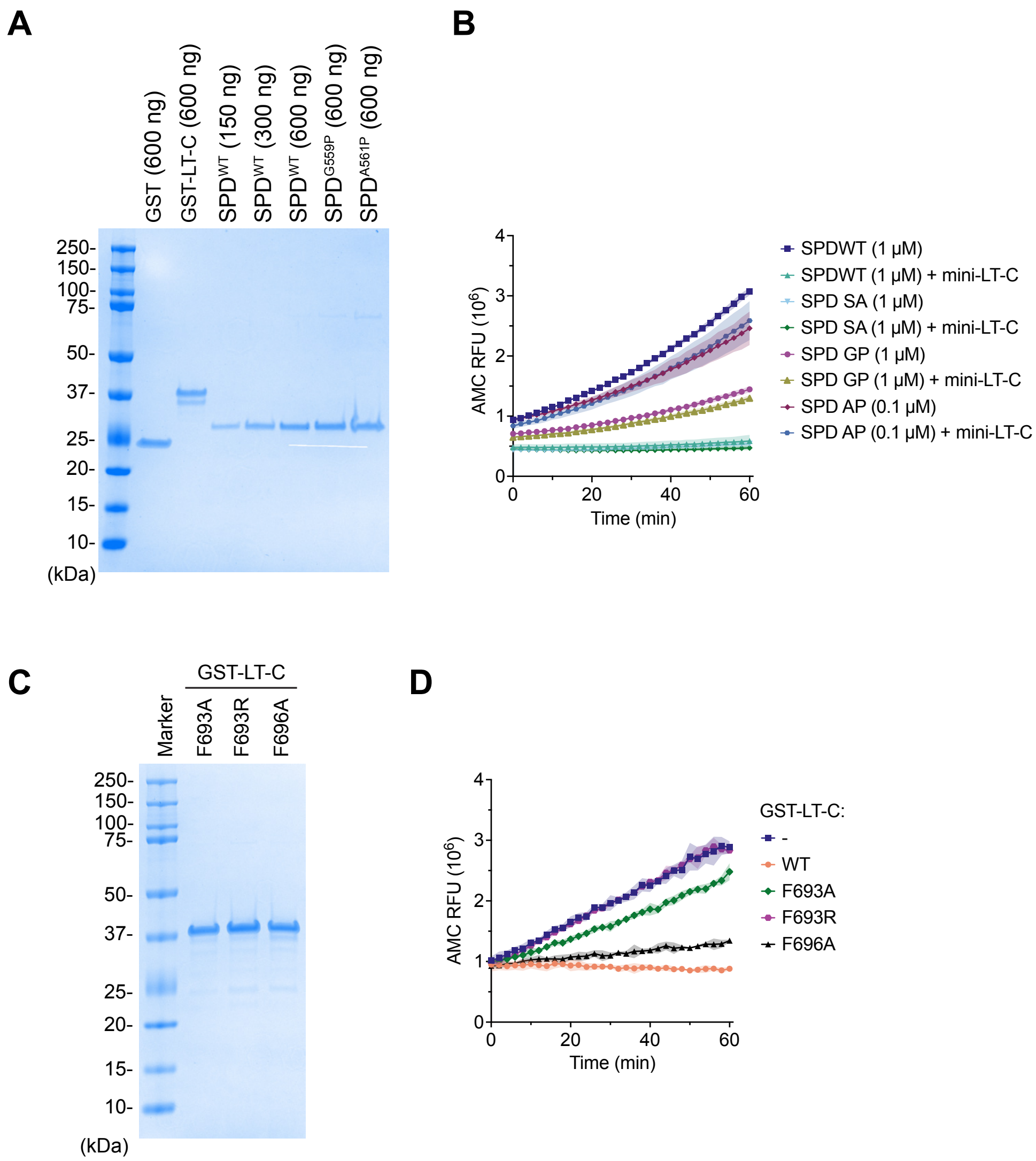

Supplementary Figure 6

bioRxiv preprint doi: https://doi.org/10.64898/2026.08.07.743322; this version posted August 10, 2026. The copyright holder for this preprint (which was not certified by peer review) is the author/funder, who has granted bioRxiv a license to display the preprint in perpetuity. It is made available under a CC-BY 4.0 International license.

Supplementary Figure 6. Contributions of the β10 interface and the P1-like phenylalanine residue to LT-C-mediated FAM111A inhibition.

(A) The indicated amounts of purified recombinant GST, GST-LT-C, and SPD (wild-type, G559P, and A561P) proteins analyzed by SDS-PAGE and visualized by Coomassie staining. Molecular weight markers are shown on the left.

(B) Time course of *in vitro* activity of FAM111A SPD (wild-type, G559P, and A561P) in the absence or presence of mini-LT-C. Peptidase activity was measured using Suc-AAPF-AMC as a substrate and fluorescence from released AMC was measured over time for 1 hr at 37°C. Values represent the mean of three technical replicates, and standard deviation is indicated by the shaded area. RFU: relative fluorescence units.

(C) One microgram of purified recombinant GST-LT-C mutants (F693A, F693R, and F696A) analyzed by SDS-PAGE and visualized by Coomassie staining. Molecular weight markers are shown on the left.

(D) Time course of *in vitro* FAM111A SPD activity in the presence of GST-LT-C (wild-type or phenylalanine mutants F693A, F693R, and F696A). Reactions without GST-LT-C contain GST as a control. Peptidase activity was measured as in (B). Values represent the mean of three technical replicates, and standard deviation is indicated by the shaded area.

bioRxiv preprint doi: https://doi.org/10.64898/2026.08.07.743322; this version posted August 10, 2026. The copyright holder for this preprint (which was not certified by peer review) is the author/funder, who has granted bioRxiv a license to display the preprint in perpetuity. It is made available under a CC-BY 4.0 International license.

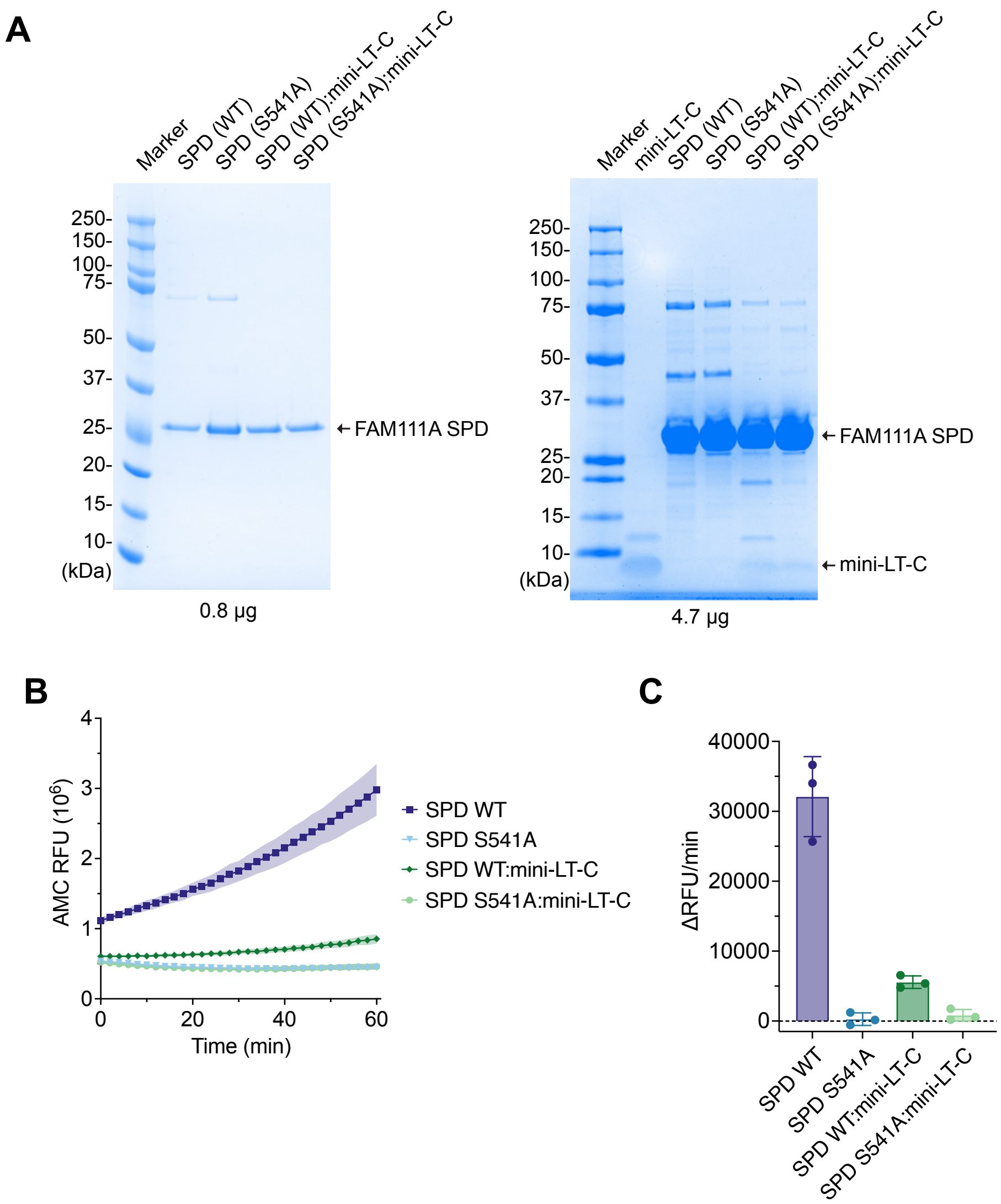

Supplementary Figure 7

bioRxiv preprint doi: https://doi.org/10.64898/2026.08.07.743322; this version posted August 10, 2026. The copyright holder for this preprint (which was not certified by peer review) is the author/funder, who has granted bioRxiv a license to display the preprint in perpetuity. It is made available under a CC-BY 4.0 International license.

Supplementary Figure 7. Purification and peptidase activity of SPD:mini-LT-C complexes.

(A) SDS-PAGE analysis of purified mini-LT-C, FAM111A SPD (wild-type and S541A), and size-exclusion chromatography-purified complexes of SPD (wild-type or S541A) with mini-LT-C. Molecular weight markers are shown on the left.

(B) Time course of FAM111A SPD activity for SPD alone (wild-type) and for purified SPD-mini-LT-C complexes (wild-type or S541A). Peptidase activity was measured using Suc-AAPF-AMC as a substrate and fluorescence from released AMC was measured over time for 1 hr at 37°C. Values represent the mean of three technical replicates, and standard deviation is indicated by the shaded area. RFU: relative fluorescence units.

(C) Peptidase activity of SPD expressed as change in relative fluorescence units per minute (ΔRFU/min). Values are mean ± SD of three technical replicates.

bioRxiv preprint doi: https://doi.org/10.64898/2026.08.07.743322; this version posted August 10, 2026. The copyright holder for this preprint (which was not certified by peer review) is the author/funder, who has granted bioRxiv a license to display the preprint in perpetuity. It is made available under a CC-BY 4.0 International license.

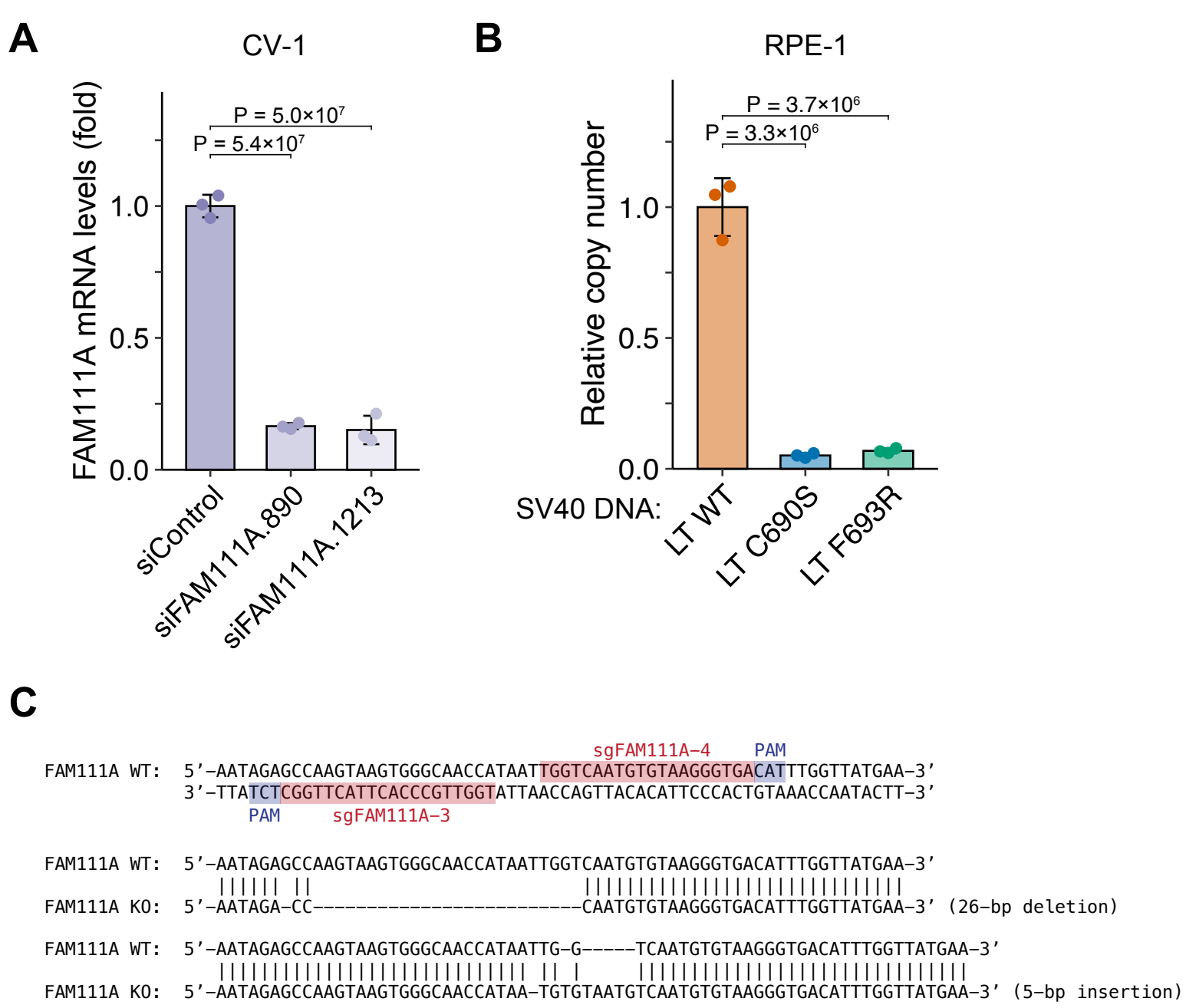

Supplementary Figure 8

bioRxiv preprint doi: https://doi.org/10.64898/2026.08.07.743322; this version posted August 10, 2026. The copyright holder for this preprint (which was not certified by peer review) is the author/funder, who has granted bioRxiv a license to display the preprint in perpetuity. It is made available under a CC-BY 4.0 International license.

Supplementary Figure 8. Validation of FAM111A knockdown and knockout.

(A) Relative FAM111A mRNA levels in CV-1 cells transfected with control siRNA (siControl) or two independent siRNAs targeting FAM111A. mRNA levels were measured by quantitative PCR, normalized to GAPDH, and expressed relative to the control sample. Data represent mean ± SD of three biological replicates. Statistical significance was determined by one-way ANOVA with Dunnett's multiple-comparison test.

(B) Quantification of SV40 genome copy number in RPE-1 cells transfected with WT, C690S, F693R SV40 genomic DNA. Viral genomic copy number was determined by quantitative PCR 72 hr after transfection. Values are mean ± SD of three technical replicates. Statistical significance was determined by one-way ANOVA with Dunnett's multiple-comparison test.

(C) Schematic of sgRNA target sites within FAM111A exon 4 showing the positions of sgFAM111A-3 and sgFAM111A-4 and their PAM sequences. Sequence alignments of wild-type (WT) and knockout (KO) alleles are shown. Sizes of each insertion and deletion at the target sites are indicated.

bioRxiv preprint doi: https://doi.org/10.64898/2026.08.07.743322; this version posted August 10, 2026. The copyright holder for this preprint (which was not certified by peer review) is the author/funder, who has granted bioRxiv a license to display the preprint in perpetuity. It is made available under a CC-BY 4.0 International license.

Supplementary Table 1. X-ray crystallographic data collection and refinement statistics

	SPD^{VDVD} apo	SPD^{VDVD}:mini-LT-C
**Data collection**		
Space group	$p2_12_12$	$p2_1$
Cell dimensions		
a, *b*, *c* (Å)	74.86, 57.65, 65.22	72.44 ,57.45 ,73.69
α, β, γ (°)	90, 90, 90	90.00, 92.49, 90.00
Resolution (Å)	50-1.85 (1.92-1.85)	50.00-1.66 (1.72-1.66)
R_{sym} or R_{merge}	0.130 (0.788)	0.131 (0.967)
$I / \sigma I$	13.5 (1.8)	15.8 (2.0)
Completeness (%)	97.5 (91.2)	98.4 (92.5)
Redundancy	5.3 (3.6)	8.8 (6.4)
**Refinement**		
Resolution (Å)	50-1.85	45.30 - 1.66
No. reflections	23764	70494
R_{work} / R_{free}	0.156/0.191	0.168/0.209
No. atoms		
Protein	2066	4421
Ligand/ion	6	34
Water	214	387
B-factors		
Protein	29.15	37.2
Ligand/ion	34.8	51.4
Water	37.7	41.5
R.m.s. deviations		
Bond lengths (Å)	0.003	0.020
Bond angles (°)	0.63	1.49

Values in parentheses refer to the highest resolution shell (10% of reflections)

bioRxiv preprint doi: https://doi.org/10.64898/2026.08.07.743322; this version posted August 10, 2026. The copyright holder for this preprint (which was not certified by peer review) is the author/funder, who has granted bioRxiv a license to display the preprint in perpetuity. It is made available under a CC-BY 4.0 International license.

Supplementary Table 2. Gene fragments used in this study

Gene fragment name	Sequence	Length (bp)
LT 665-708	TCTGTTCCAGGGGCCCCTGGGATCCCAGTCCCAA GGCTCATTTCAGGCCCCTCAGTCCTCACAGTCTGT TCATGATCATAATCAGCCATACCACATTTGTAGAG GTTTTACTTGCTTTAAAAAACCTCCCACACCTCCCC CTGAACCTGAAACATAAGCGGCCGCATCGTGACTG ACTGACGA	183
LT 674-708	TCTGTTCCAGGGGCCCCTGGGATCCCCTCAGTCCT CACAGTCTGTTCATGATCATAATCAGCCATACCACA TTTGTAGAGGTTTTACTTGCTTTAAAAAACCTCCCAC ACCTCCCCCTGAACCTGAAACATAAGCGGCCGCATC GTGACTGACTGACGA	159
LT 679-708	TCTGTTCCAGGGGCCCCTGGGATCCGTTCATGATCA TAATCAGCCATACCACATTTGTAGAGGTTTTACTTGC TTTAAAAAACCTCCCACATAAGAATTCCCGGGTCGAC TCGAGCGGCCGCATCGTGACTGACTGACGA	140

Supplementary Table 3. Oligonucleotides and guide RNAs used in this study

Target gene	Oligo name	Sequence (5′ → 3′)	Type	Application
Large T	LT 627_Bam For	GCATGGATCCGATTGGCTAAG AAACAGTGATG	Primer	Cloning
Large T	LT654_Bam For	GCATGGATCCATGGAAGACTC AGGGCATGAAACAG	Primer	Cloning
Large T	LT 708_Not Rev	GCATGCGGCCGCTTATGTTTC AGGTTCAGGGGGAGGTG	Primer	Cloning
Large T	LT_F693A-C Rev	CGATGCGGCCGCTTATGTTTC AGGTTCAGGGGGAGGTGTGG GAGGTTTTTTAAAGCAAGTAG CACCTCTACAAATGTGG	Primer	Cloning
Large T	LT_H681N For	CAGTCCTCACAGTCTGTTAAT GATCATAATCAGCCATACC	Primer	Mutagenesis
Large T	LT_H681N Rev	GGTATGGCTGATTATGATCATT AACAGACTGTGAGGACTG	Primer	Mutagenesis
Large T	LT_H681N_H683N_Fwd_COcoli	CACAGTCTGTTaATGATaATAA TCAGCCATACCACATTTG	Primer	Mutagenesis
Large T	LT_H681N_H683N_Rev_COcoli	CAAATGTGGTATGGCTGATTA TtATCATtAACAGACTGTG	Primer	Mutagenesis
Large T	LT_D682A_Fwd_COcoli	CACAGTCTGTTCATGcTCATAA TCAGCCATAC	Primer	Mutagenesis

bioRxiv preprint doi: https://doi.org/10.64898/2026.08.07.743322; this version posted August 10, 2026. The copyright holder for this preprint (which was not certified by peer review) is the author/funder, who has granted bioRxiv a license to display the preprint in perpetuity. It is made available under a CC-BY 4.0 International license.

Large T	LT_D682A_Rev_COcoli	GTATGGCTGATTATGAgCATGAACAGACTGTG	Primer	Mutagenesis
Large T	LT_H683N For	CACAGTCTGTTCATGATAATAATCAGCCATACCACATTTG	Primer	Mutagenesis
Large T	LT_H683N Rev	CAAATGTGGTATGGCTGATTATTATCATGAACAGACTGTG	Primer	Mutagenesis
Large T	LT_H688N For	GATCATAATCAGCCATACAACATTTGTAGAGGTTTTAC	Primer	Mutagenesis
Large T	LT_H688N Rev	GTAAAACCTCTACAAATGTTGTATGGCTGATTATGATC	Primer	Mutagenesis
Large T	LT_C690S For	CATAATCAGCCATACCACATTTCTAGAGGTTTTACTTGC	Primer	Mutagenesis
Large T	LT_C690S Rev	GCAAGTAAAACCTCTAGAAATGTGGTATGGCTGATTATG	Primer	Mutagenesis
Large T	LT_F693R For	CCACATTTGTAGAGGTCGTACTTGCTTTAAAAAACC	Primer	Mutagenesis
Large T	LT_F693R Rev	GGTTTTTTAAAGCAAGTACGACCTCTACAAATGTGG	Primer	Mutagenesis
Large T	LT_C695S For	CATTTGTAGAGGTTTTACTTCCTTTAAAAAACCTCCCAC	Primer	Mutagenesis
Large T	LT_C695S Rev	GTGGGAGGTTTTTTAAAGGAAGTAAAACCTCTACAAATG	Primer	Mutagenesis
Large T	LT_F696A For	GTAGAGGTTTTACTTGCGCTAAAAAACCTCCCACAC	Primer	Mutagenesis
Large T	LT_F696A Rev	GTGTGGGAGGTTTTTTAGCGCAAGTAAAACCTCTAC	Primer	Mutagenesis
Large T	LT_RTqPCR_MC_F	AAGGCATTCCACCACTGCTC	Primer	qRT-PCR
Large T	LT_RTqPCR_MC_R	ACCTGACTTTGGAGGCTTCTG	Primer	qRT-PCR
VP1	VP1_RTqPCR_MC_F1	TTGGTGGGGAACCTTTGGAG	Primer	qRT-PCR
VP1	VP1_RTqPCR_MC_R1	GGATCAGGAACCCAGCACTC	Primer	qRT-PCR
VP2/VP3	SV40_VP23_Taq_SL1_Fw	GCACATTAGGACTTTGGCTTTGA (Ref #41)	Primer	qRT-PCR; qPCR (genome copy number)
VP2/VP3	SV40_VP23_Taq_SL1_Rev	CAGGGAAGGGTTGCAAATATCA (Ref #41)	Primer	qRT-PCR; qPCR (genome copy number)

bioRxiv preprint doi: https://doi.org/10.64898/2026.08.07.743322; this version posted August 10, 2026. The copyright holder for this preprint (which was not certified by peer review) is the author/funder, who has granted bioRxiv a license to display the preprint in perpetuity. It is made available under a CC-BY 4.0 International license.

FAM111A (codon-optimized)	hFAM111A-2VD-FOR	CGATTAAAGTAGATAAACTTCTTGATCGTCTCAGTGAC	Primer	Mutagenesis
FAM111A (codon-optimized)	hFAM111A-2VD-REV	GTCACTGAGACGATCAAGAAGTTTATCTACTTTAATCG	Primer	Mutagenesis
FAM111A (codon-optimized)	SPD-A561P-Fp	GCATGCAGCAGGTTTTCCGTATACCTATCAGAATG	Primer	Mutagenesis
FAM111A (codon-optimized)	SPD-A561P-Rp	CATTCTGATAGGTATACGGAAAACCTGCTGCATGC	Primer	Mutagenesis
FAM111A (codon-optimized)	SPD-G559P-Fp	GCAATGCATGCAGCACCGTTTGCATATACCTATC	Primer	Mutagenesis
FAM111A (codon-optimized)	SPD-G559P-Rp	GATAGGTATATGCAAACGGTGCTGCATGCATTGC	Primer	Mutagenesis
FAM111A	FAM111A-SR890-For	CGAAAACGACGACTGGAAGCTCATTGAAAACAATGACACC	Primer	Mutagenesis
FAM111A	FAM111A-SR890-Rev	CTTCCAGTCGTCGTTTTCGAGAAAGGAAAGAAATCTGCCATC	Primer	Mutagenesis
FAM111A	CsFAM111A_Ex3_F1	GGTCACAGAAGCACTCAGTCA	Primer	qRT-PCR
FAM111A	CsFAM111A_Ex4_R3	TTTGGGCATAGTCTGGTCTTC	Primer	qRT-PCR
FAM111A	FAM111A Ex4 F2	ACAGTGCAACTACGGGTTACG	Primer	Genotyping PCR
FAM111A	FAM111A Ex4 R2	TTCAGGACAGCATAGTCAAGC	Primer	Genotyping PCR
GAPDH	CsGAPDH_1F	CAATGACCCCTTTATTGACC	Primer	qRT-PCR
GAPDH	GAPDH 1R	GACAAGCTTCCCGTTCTCAG	Primer	qRT-PCR
Non-targeting	siControl	CGUACGCGGAAUACUUCGA	siRNA	Knockdown control
FAM111A	siFAM111A.890	GGAGAAUGAUGAUUGGAAA	siRNA	Gene knockdown
FAM111A	siFAM111A.1213	CGAUUAAAGUAGUGAAACU	siRNA	Gene knockdown
FAM111A	sgFAM111A-3	ATTATGGTTGCCCACTTACT	sgRNA	CRISPR
FAM111A	sgFAM111A-4	CAATGTGTAAGGGTGACATT	sgRNA	CRISPR

bioRxiv preprint doi: https://doi.org/10.64898/2026.08.07.743322; this version posted August 10, 2026. The copyright holder for this preprint (which was not certified by peer review) is the author/funder, who has granted bioRxiv a license to display the preprint in perpetuity. It is made available under a CC-BY 4.0 International license.

Supplementary Table 4. Plasmids used in this study

Plasmid name	Backbone	Insert / description	Tag	Source
pDB.His.MBP/FAM111A SPD (WT; codon-optimized)	pDB.His.MBP	Human FAM111A SPD (aa 335-611)	His_6-MBP	Ref #22
pDB.His.MBP/FAM111A SPD (S541A; codon-optimized)	pDB.His.MBP	Catalytically inactive FAM111A SPD (S541A)	His_6-MBP	Ref #22
pDB.His.MBP/FAM111A mini-SPD (codon-optimized)	pDB.His.MBP	Dimerization site mutant of FAM111A SPD	His_6-MBP	Ref #22
pDB.His.MBP/FAM111A SPD (VDVD; codon-optimized)	pDB.His.MBP	Dimerization site mutant of FAM111A SPD	His_6-MBP	Ref #22
pDB.His.MBP/FAM111A SPD (G556P; codon-optimized)	pDB.His.MBP	β10 mutant of FAM111A SPD	His_6-MBP	This study
pDB.His.MBP/FAM111A SPD (A561P; codon-optimized)	pDB.His.MBP	β10 mutant of FAM111A SPD	His_6-MBP	This study
pGEX-6P-2/LT-C (WT)	pGEX-6P-2	SV40 Large T C-terminal region (aa 627-708)	GST	This study
pGEX-6P-2/LT-C (H681N)	pGEX-6P-2	LT-C H681N mutant	GST	This study
pGEX-6P-2/LT-C (H681N/H683N)	pGEX-6P-2	LT-C double mutant H681N/H683N	GST	This study
pGEX-6P-2/LT-C (D682A)	pGEX-6P-2	LT-C D682A mutant	GST	This study
pGEX-6P-2/LT-C (H683N)	pGEX-6P-2	LT-C H683N mutant	GST	This study
pGEX-6P-2/LT-C (H688N)	pGEX-6P-2	LT-C H688N mutant	GST	This study
pGEX-6P-2/LT-C (C690S)	pGEX-6P-2	LT-C C690S mutant	GST	This study
pGEX-6P-2/LT-C (F693A)	pGEX-6P-2	LT-C F693A mutant	GST	This study
pGEX-6P-2/LT-C (F693R)	pGEX-6P-2	LT-C F693R mutant	GST	This study
pGEX-6P-2/LT-C (C695S)	pGEX-6P-2	LT-C C695S mutant	GST	This study
pGEX-6P-2/LT-C (F696A)	pGEX-6P-2	LT-C F696A mutant	GST	This study
pGEX-6P-2/LT (654-708)	pGEX-6P-2	SV40 Large T (aa 654-708)	GST	This study
pGEX-6P-2/LT (665-708)	pGEX-6P-2	SV40 Large T (aa 665-708)	GST	This study
pGEX-6P-2/LT (674-708)	pGEX-6P-2	SV40 Large T (aa 674-708)	GST	This study
pGEX-6P-2/mini-LT-C (WT)	pGEX-6P-2	SV40 Large T (aa 679-708)	GST	This study
pUC18/SV40 (WT)	pUC18	SV40 genome (strain 776)	N/A	This study

bioRxiv preprint doi: https://doi.org/10.64898/2026.08.07.743322; this version posted August 10, 2026. The copyright holder for this preprint (which was not certified by peer review) is the author/funder, who has granted bioRxiv a license to display the preprint in perpetuity. It is made available under a CC-BY 4.0 International license.

pUC18/SV40 (C690S)	pUC18	SV40 genome encoding LT C690S	N/A	This study
pUC18/SV40 (F693R)	pUC18	SV40 genome encoding LT F693R	N/A	This study
pLVX2-IRES-Puro/ FAM111A SR890 (WT)	pLVX2-IRES-Puro	Full-length human FAM111A (resistant to siFAM111A.890)	N/A	This study
pLVX2-IRES-Puro/ FAM111A SR890 (S541A)	pLVX2-IRES-Puro	Catalytically inactive FAM111A (resistant to siFAM111A.890)	N/A	This study
pLVX2-IRES-Puro/ FAM111A SR890 (YFAA)	pLVX2-IRES-Puro	PIP motif mutant (YFAA) (resistant to siFAM111A.890)	N/A	This study
pMD2.G	N/A	Lentiviral packaging plasmid	N/A	Addgene #12259
psPAX2	N/A	Lentiviral envelope plasmid	N/A	Addgene #12260

Supplementary Table 5. Synthetic peptides used in this study

Peptide name	Sequence	Length (aa)	Modification
core-LT-C (WT)	SVHDHNQPYHICRGFTCFKKPPTPPP	26	None
core-LT-C (C690S)	SVHDHNQPYHISRGFTCFKKPPTPPP	26	None
core-LT-C-biotin (WT)	SVHDHNQPYHICRGFTCFKKPPTPPPK	27	C-terminal Lys-biotin
core-LT-C-biotin (C690S)	SVHDHNQPYHISRGFTCFKKPPTPPPK	27	C-terminal Lys-biotin
FITC-core-LT-C (WT)	SVHDHNQPYHICRGFTCFKKPPTPPP	26	N-terminal FITC-Ahx

Supplementary Table 6. Antibodies used in this study

Target protein	Application	Host species	Source	Catalog number	Dilution for western blot
FAM111A	Western blot	Rabbit	Abcam	ab184572	1:1,000
FAM111A	Western blot	Rabbit	Sigma	HPA040176	1:1,000
FAM111A (SPD)	Western blot	Rabbit	Cocalico (custom)	N/A	1:1,000
SV40 Large T antigen	Western blot, Immunoprecipitation	Mouse	Abcam	ab16879	1:2,000
SV40 VP1	Western blot	Rabbit	Abcam	ab53977	1:2,000

bioRxiv preprint doi: https://doi.org/10.64898/2026.08.07.743322; this version posted August 10, 2026. The copyright holder for this preprint (which was not certified by peer review) is the author/funder, who has granted bioRxiv a license to display the preprint in perpetuity. It is made available under a CC-BY 4.0 International license.

SV40 VP2/3	Western blot	Rabbit	Abcam	ab53983	1:1,000
GAPDH	Western blot	Mouse	GeneTex	GTX627408	1:1,000
